# Cluster-Based Analysis of Infectious Disease Occurrences Using Tensor Decomposition: A Case Study of South Korea

**DOI:** 10.3390/ijerph17134872

**Published:** 2020-07-06

**Authors:** Seungwon Jung, Jaeuk Moon, Eenjun Hwang

**Affiliations:** School of Electrical Engineering, Korea University, 145 Anam-ro, Seongbuk-gu, Seoul 02841, Korea; jsw161@korea.ac.kr (S.J.); jaewookmo@korea.ac.kr (J.M.)

**Keywords:** tensor decomposition, infectious disease occurrence, pattern analysis, clustering

## Abstract

For a long time, various epidemics, such as lower respiratory infections and diarrheal diseases, have caused serious social losses and costs. Various methods for analyzing infectious disease occurrences have been proposed for effective prevention and proactive response to reduce such losses and costs. However, the results of the occurrence analyses were limited because numerous factors affect the outbreak of infectious diseases and there are complex interactions between these factors. To alleviate this limitation, we propose a cluster-based analysis scheme of infectious disease occurrences that can discover commonalities or differences between clusters by grouping elements with similar occurrence patterns. To do this, we collect and preprocess infectious disease occurrence data according to time, region, and disease. Then, we construct a tensor for the data and apply Tucker decomposition to extract latent features in the dimensions of time, region, and disease. Based on these latent features, we conduct k-means clustering and analyze the results for each dimension. To demonstrate the effectiveness of this scheme, we conduct a case study on data from South Korea and report some of the results.

## 1. Introduction

Historically, infectious diseases have had devastating consequences for public health. For instance, according to the report published in 2018 by the World Health Organization (WHO) [1], three infectious diseases: lower respiratory infection, diarrheal disease, and tuberculosis were ranked in the top 10 causes of death worldwide. Further, although human immunodeficiency virus infection and acquired immune deficiency syndrome (HIV/AIDS) and malaria are not listed in the top 10, they have also caused numerous deaths. Infectious diseases have resulted in not only losses of lives but also serious social losses [2,3]. For instance, recent infectious diseases, such as Middle East respiratory syndrome (MERS), Zika virus infection, and coronavirus disease 2019 (COVID-19), have had high infection rates, significant mortality rates, and severe aftereffects. As soon as their outbreaks were reported, most economic and social activities were restricted due to the fear of infection, resulting in serious social losses and costs.

To reduce such losses and costs, most countries have established national health institutes and have carried out diverse activities, such as disinfection, vaccination, campaigns, and quarantines. One critical factor necessary to improve the effectiveness of such activities is to analyze the previous occurrences of infectious diseases [4]. Based on the analysis results, governments and national health institutes in various countries can predict disease occurrences in the near future and take measures to reduce the risk of the expected infectious diseases, which includes vaccine production, effective regulations, and prevention campaigns. Hence, a variety of methods from statistical approaches to machine learning-based approaches have been used to analyze the occurrence of infectious diseases [5,6,7]. One representative goal in the analysis was to reveal the relationships between infectious diseases and factors in diverse fields, such as meteorology, sociology, and geography. The results of the analysis could be used as a basis to select crucial factors or eliminate extraneous factors when predicting the occurrence of infectious diseases, improving prediction performance [8].

Rodó et al. [9] introduced several studies on disease prediction models using climate data and analyzed the effects of the climate on infectious disease occurrences. In addition, they indicated the need for a sophisticated climate model suitable for future climate changes to ensure the performance of the prediction models. Vazquez-Prokopec et al. [10] collected global positioning system (GPS) data and infectious disease occurrence data on citizens and constructed a model based on the data to determine the relationship between them. They proposed a few basic rules regarding human mobility and, using a case study, demonstrated that understanding individual movement patterns is critical in infectious disease dynamics. Goscé et al. [11] analyzed the relationship between public transportation and infectious disease occurrence in cities. They concluded that public transportation of citizens is associated with infectious disease transmission. Further, Grassly and Fraser [12] examined the causes and consequences of seasonality. They derived several results concerning the interpretation of disease occurrence data, such as the association of transmission mechanisms and their transmission routes, the effects of seasonality on disease occurrences, and mathematical analyses of vaccination programs.

However, the results of previous analytical studies on infectious disease occurrences are not yet sufficient. This is because it is challenging to evaluate the extent to which various factors known to be associated with the development of an epidemic, such as environment, culture, or climate [13], influenced the occurrence of a particular epidemic. Further, even with the same disease, the influence of these factors may vary depending on spatial conditions, such as the region or country, and temporal conditions. For instance, in temperate countries, influenza is correlated with changes in temperature and absolute humidity but exhibits less correlation in tropical countries [14]. Moreover, the influence of climate on infectious disease occurrences gradually varies according to global climate changes [15].

A clustering-based approach can be used to solve the aforementioned problems. Clustering involves grouping of similar elements of a given set of elements. By analyzing the clusters, we discover common or discriminative factors among the clusters that are likely to affect disease occurrence patterns. This approach has been applied in various fields, including business, education, and biology [16,17,18]. Further, to analyze infectious diseases, several studies based on this approach have been reported. For instance, Xiao et al. [19] collected individual contact data from a survey and grouped the individuals into clusters using the *k*-medoids clustering algorithm to explore whether clusters of contacts could better explain the transmission of infectious diseases. They demonstrated that their methodology could provide insight into the structures underlying infection transmission, particularly the role of age-assortative contacts. Sloan et al. [20] presented a clustering-based analysis method using a spatial scan statistic and spatiotemporal wavelet analysis to discover how local socioeconomic factors influence both the timing and intensity of influenza and concluded that socioeconomic factors heavily affect local patients with influenza. McCloskey and Poon [21] presented a method to identify potential outbreaks of infectious diseases based on clustering in the genetic sequences and evaluated their method using both simulated and actual HIV sequence datasets. Guilamet et al. [22] applied a cluster analysis to variables from patient characteristics, acuity of illness/clinical presentation, and infection characteristics to identify determinants associated with bloodstream infection.

The results of clustering are highly dependent on the features used. Unlike the aforementioned work, we use infectious disease occurrence data as features to group elements with similar occurrence patterns. More specifically, we arrange the data by time, region, and infectious disease to analyze infectious disease occurrences effectively. However, rather than using them as they are, we extract latent features from the data and exploit them for clustering, which leads to a fast, robust, and general analysis [23,24,25]. This can be done easily by organizing data into tensors, decomposing them for feature extraction, and clustering the extracted latent features.

To demonstrate the effectiveness of the proposed scheme, we conduct a case study using the infectious disease occurrence data provided by the Infectious Diseases Portal [26] of the Korea Centers for Disease Control and Prevention (KCDC) in South Korea.

The contributions of the paper are summarized as follows:

1. We propose an analysis scheme for infectious disease occurrences based on the Tucker decomposition and *k*-means clustering by identifying elements with similar patterns of disease occurrence in terms of time, region, and disease.
2. We show how to interpret the commonalities and differences between clusters in terms of time, region, and disease. By doing so, we can discover possible factors that can affect the pattern of disease occurrences.
3. We reveal the effectiveness of our scheme by conducting a case study on the infectious disease occurrence patterns in South Korea.

The rest of the paper is organized as follows. We describe the clustering-based analysis scheme based on the Tucker decomposition and *k*-means clustering in Section 2. We demonstrate and discuss the analytical results in Section 3. Finally, we present the conclusions in Section 4.

## 2. Methods

**Figure 1:**
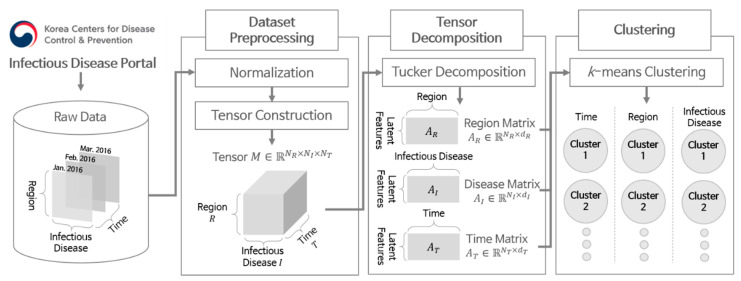
Overall steps for data analysis.

Figure 1 illustrates the overall flow of the proposed scheme. The scheme consists of three main steps: dataset preprocessing, tensor decomposition, and clustering. We first describe how to collect the dataset of disease occurrences and preprocess them in Section 2.1. Then, we explain how to decompose the data using tensors and perform clustering in Section 2.2 and Section 2.3, respectively.

### 2.1. Dataset Collection and Preprocessing

**Figure 2:**
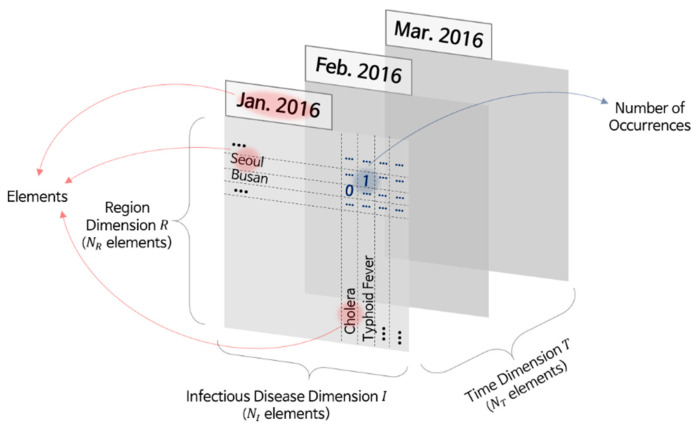
Disease occurrence data organized by date.

The infectious disease occurrence data we used for analysis contained data on the infectious disease, region, and number of reported patients by date. For instance, in January 2016, the number of patients with cholera in Busan was zero, the number of patients with typhoid fever in Seoul was one, and the number of patients with mumps in Gyeonggi-do was 216. We focused on three elements: region, time, and infectious disease denoted as the *R*, *T*, and *I* dimensions, respectively. Further, we denoted the number of elements in each dimension by *N_R_*, *N_T_*, and *N_I_*, respectively. Figure 2 illustrates the occurrence data organized by place and disease on a specific date in the three-dimensional (3D) space.

In this paper, we used the infectious disease occurrence data from South Korea, provided by the Infectious Diseases Portal [26] of the KCDC. This dataset is a collection of the number of newly reported patients for 59 infectious diseases every month. The numbers were based on the patients reported to public health agencies in 17 regions, and the occurrence region was determined based on the patients’ addresses. We collected the occurrence data from January 2016 to October 2019 and removed missing values in the collected data. The missing values were due to the change in the legal infectious diseases list for South Korea. Because KCDC focuses on monitoring the occurrence of legal infectious diseases, the disease occurrences before the designation were not provided, and their counts were zero in the Infectious Diseases Portal. We deleted the infectious disease data containing missing values, and as a result, 56 infectious diseases remained. The infectious diseases and regions contained in the dataset are listed in Table 1 and Table 2, respectively.

For the collected occurrence data, we performed normalization on the number of patients with infectious diseases to reduce the effect of more common infectious diseases that many patients have developed. Without normalization, the analysis results significantly depend on those diseases, while the other diseases have a trivial effect. However, some diseases that had few patients had a high mortality rate or high contagion. For instance, MERS is a rarely reported infectious disease in South Korea. However, when the outbreak of MERS was reported in 2015, it caused about 38 fatalities and a tremendous amount of economic damage in South Korea [27]. Therefore, we performed normalization because it was necessary to prevent the analysis results from being too dependent on a few specific diseases.

**Figure 3:**
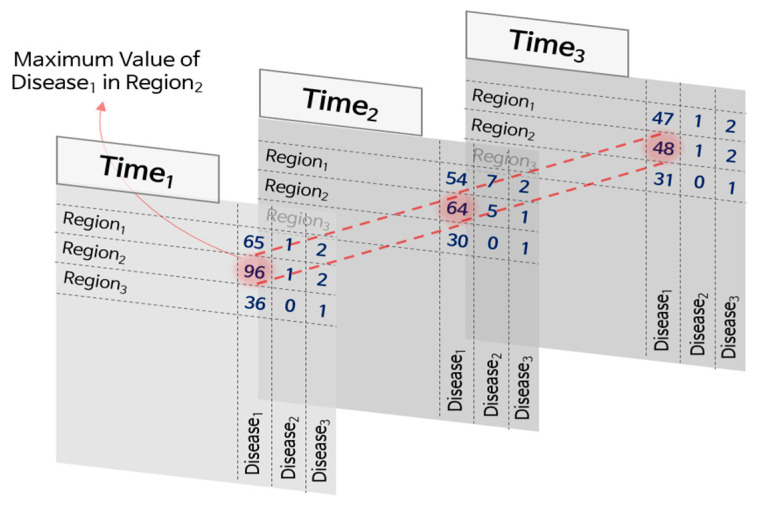
Normalization of the number of disease patients.

For normalization, we first determined the maximum number of patients during the period for each disease and region pair and then divided the number of patients by the maximum value. During the normalization process, the occurrence values in the dataset were converted into real numbers between zero and one. Figure 3 illustrates this process. In the figure, the occurrence values of 96, 64, and 48 for the pair *Region*_1_ and *Disease*_2_ were converted into 1.0, 0.67, and 0.5, respectively, after normalization, as the maximum value is 96.

More specific reasons for using normalization are the following. We can reduce the influence of diseases that have significantly more patients compared to other diseases, as mentioned above. If we normalize the number of patients without considering their relative differences, the problem remains unsolved. Thus, we used the maximum patient number for each infectious disease. The normalized values of one disease are determined only by the maximum number of patients with the disease, no matter how many patients are infected with other diseases. For instance, in Figure 3, *Disease*_1_ has more patients than *Disease*_2_ and *Disease*_3_. If we perform the normalization, *Disease*_1_ has 1.0, 0.67, and 0.5 normalized values for *Time*_1_, *Time*_2_, and *Time*_3_, respectively, in *Region*_2_, whereas *Disease*_2_ has 0.2, 1.0, and 0.2 normalized values for the same region at the respective times.

Moreover, we can suppress the effects of the difference in population between regions. The number of patients in the region tends to become larger as the population increases. Thus, dividing the data by the maximum number of patients in the region removes the effects of the population differences. Dividing the number of patients by the population might be one option. However, severe but rarely occurring diseases have few patients, while the population can be relatively numerous. Then, the normalized values become close to zero, and it is difficult to affect the analysis results.

To handle 3D data (region, infectious disease, and time) effectively, we represented the disease occurrence data using tensors. A tensor is a multidimensional array for dealing with data whose dimension is equal to or higher than three. In the case of disease occurrence data, the tensor *M* has three dimensions *R*, *I,* and *T*, and the resulting size is *N_R_* × *N_I_* × *N_T_*. The tensor contains normalized patient numbers according to *R*, *I,* and *T*.

### 2.2. Tensor Decomposition

By decomposing a tensor, we extracted diverse latent features from each tensor element. Compared with raw data-based clustering, latent feature-based clustering has the following advantages. (i) It can decrease computation time and memory requirements by reducing the data dimensionality [23]. (ii) It is also more robust to noise [24]. (iii) Finally, latent features can represent the data in a more general form than the raw data [25].

To extract latent features from the raw data, we used a tensor decomposition technique that divides a tensor into smaller tensors or matrices. Tensor decomposition has been commonly used to extract latent features from data whose form is a tensor and has demonstrated its effectiveness in data analysis [28,29]. Among various methods for tensor decomposition, we used the Tucker decomposition because it is a generalized form of tensor decomposition [30,31]. It has been widely used for latent feature extraction in diverse domains, such as vectorized electroencephalography signals [32], human behaviors [33], and drug responses to diseases [34].

Tucker decomposition divides a given tensor into four components: three matrices corresponding to each dimension and one core tensor. Equation (1) is the equation for the Tucker decomposition, and all components in the equation are generally obtained by high-order orthogonal iteration [35], where γ is a core tensor whose size is *d_R_* × *d_I_* × *d_T_*, ×*_D_* denotes a *k*-mode product for a dimension *D*, and *A_D_* is a matrix of *D* whose size is *N_D_* × *d_D_*.

(1) $$M\approx \gamma \times_{R}A_{R}\times_{I}A_{I}\times_{T}A_{T}.$$

**Figure 4:**
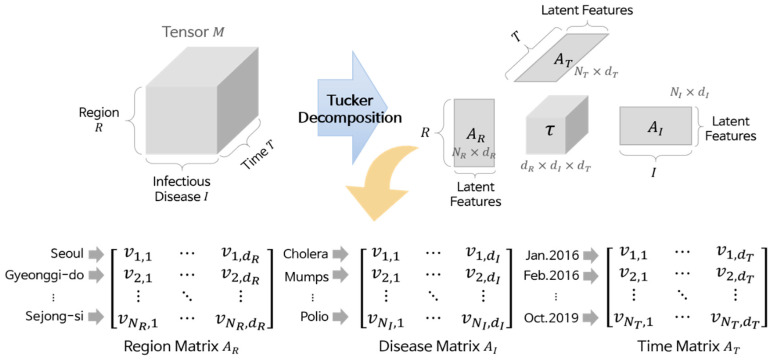
Tucker decomposition.

Figure 4 illustrates the details of the Tucker decomposition. Here, *A_D_* contains the latent features of the elements in dimension *D*, and these features consist of *d_D_* real values (i.e., each element is represented by a vector). For instance, in the region matrix *A_R_*, “Seoul” is represented by $\left[v_{1,\;1},\ldots ,v_{1,\;d_{R}}\right]$, and “Gyeonggi-do” is represented by $\left[v_{2,\;1},\ldots ,v_{2,\;d_{R}}\right]$. Similarly, “Polio” is represented by $\left[v_{N_{I},\;1},\ldots ,v_{N_{I},\;d_{I}}\right]$ in the disease matrix *A_I_*, and “Feb. 2016” is represented by $\left[v_{2,\;1},\ldots ,v_{2,\;d_{T}}\right]$ in the time matrix *A_T_*. We call these vectors element vectors. Although we cannot know what the values in the element vectors mean because they are latent features, the more similar two vectors are, the more similar their corresponding elements are. Based on this property, we performed clustering.

### 2.3. Clustering

We grouped similar elements using the matrices obtained from decomposition. For this purpose, we used *k*-means clustering, which is one of the most popular clustering algorithms. This algorithm separates a given set of data into *k* clusters based on the distance between data and the centers of the clusters. For each dimension *D*, the algorithm works as follows:

1. Acquire all element vectors in *D* from the matrix *A_D_*.
2. Set the number of clusters, *k*.
3. Generate center vectors of *k* clusters randomly.
4. For each element vector in *D*, calculate the Euclidean distances to the center vectors, and obtain the nearest center vector.
5. Assign each element vector to the cluster with the nearest center vector.
6. Recalculate the center vector of each cluster.
7. Repeat Steps 4 to 6 until no more changes occur in the cluster assignment.

Based on the results obtained using *k*-means clustering, we performed the data analysis. Compared with other clustering algorithms, *k*-means clustering is simple to implement and is suitable for low-dimensional data [36,37]. In contrast, the *k*-means clustering method requires prior knowledge about the optimal number of clusters [38], which is nearly impossible to achieve. Hence, we adopted the elbow method to estimate the optimal number of clusters [39,40]. That is, for *k* from 1 to *N_D_*/2, we iteratively ran *k*-means clustering and measured the sum of the squared error (SSE) between element vectors and their center vector. Then, we plotted a graph of SSE versus *k* and found *k* where the change in SSE value decreases considerably. This point is called an elbow point, and we used this *k* as the optimal number of clusters for *D*, *k_D_*. Hence, we obtained *k_D_* clusters as a clustering result for each dimension and analyzed these clusters.

## 3. Results

### 3.1. Experimental Setup

**Figure 5:**
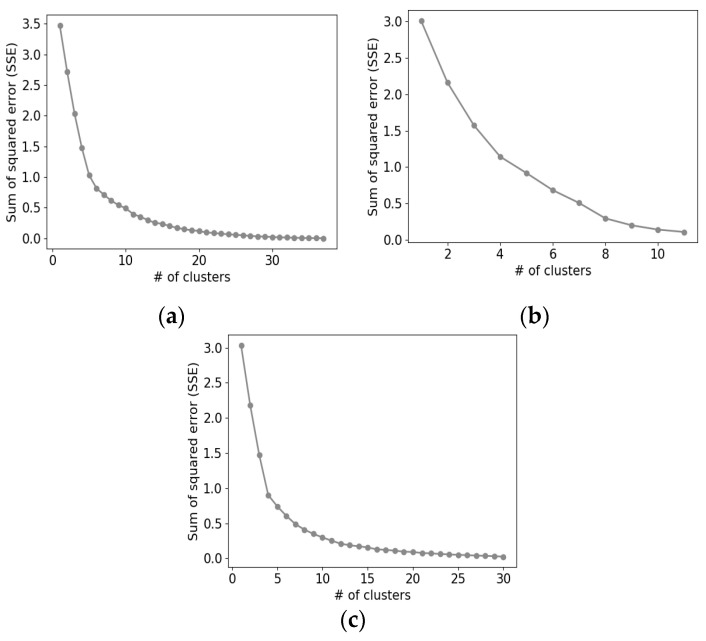
Graphs of the sum of the squared error (SSE) versus *k* for each dimension: (**a**) region, (**b**) infectious disease, and (**c**) time.

We conducted all the experiments in a Python environment. For the Tucker decomposition, we used the TensorLy [41] library, and its hyperparameters are the same as the default setting of the library except for the number of latent features. We set the number of latent features in each dimension, *d_R_*, *d_I_*, and *d_T_*, to four. To set the number of clusters for each dimension, *k_R_*, *k_I_*, and *k_T_*, we plotted graphs of SSE versus *k* for each dimension as illustrated in Figure 5 and selected six, six, and four for the optimal cluster numbers, respectively. All the hyperparameters of the proposed schemes are organized in Table 3.

From now on, we present the clustering and analytical results of the infectious disease, time, and region in turn.

### 3.2. Analysis of Disease-Based Clustering

**Figure 6:**
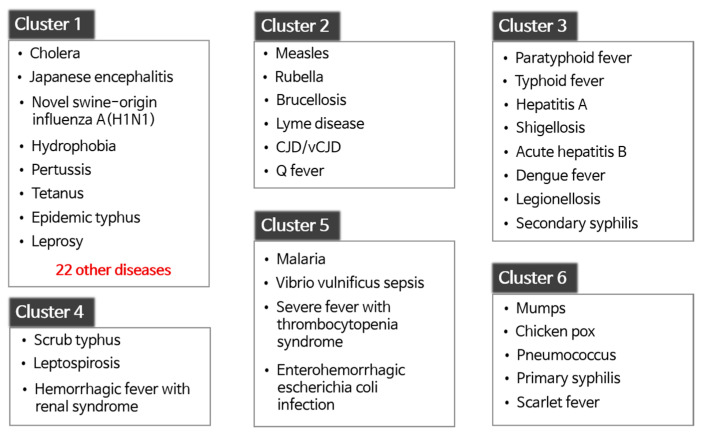
Infectious diseases in each disease cluster.

**Figure 7:**
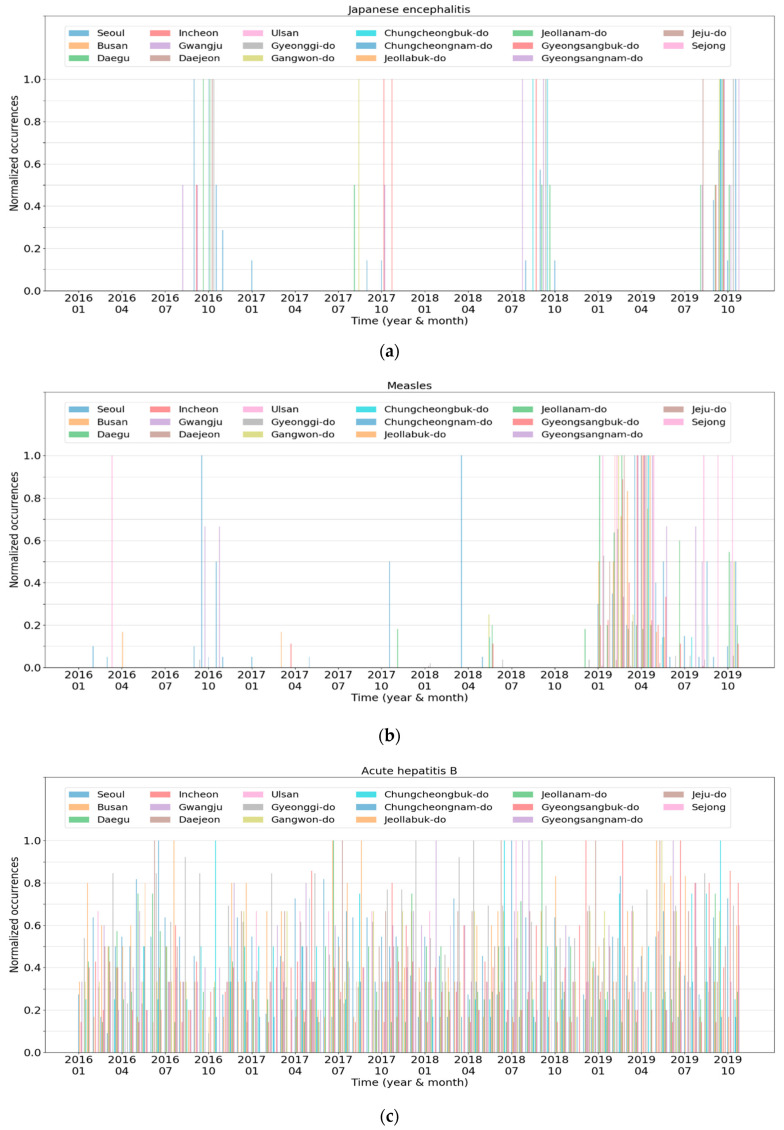

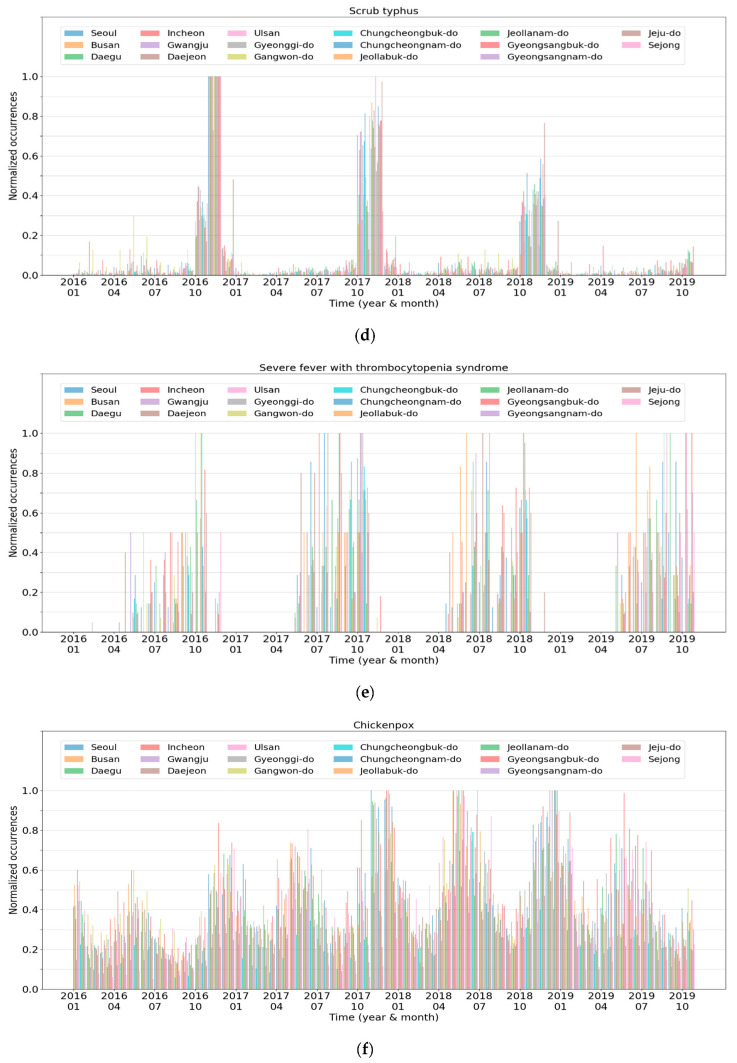
The normalized occurrences of infectious diseases in the respective regions: (**a**) Japanese encephalitis; (**b**) measles; (**c**) acute hepatitis B; (**d**) scrub typhus; (**e**) severe fever with thrombocytopenia syndrome; (**f**) chickenpox.

Figure 6 illustrates the six disease clusters obtained by clustering in terms of infectious disease. In the figure, all diseases in each cluster are listed except for Cluster 1. Because Cluster 1 contained 30 diseases, we only listed some of them. Figure 7 presents the characteristics of each cluster, which is the spatiotemporal normalized occurrences of a representative disease in each cluster. In the graphs, the *x*-axis and *y*-axis indicate the time and normalized occurrences, respectively. In addition, the 17 lines in the graphs represent the regions contained in the data.

Cluster 1 contained the 30 most infectious diseases, including cholera, Japanese encephalitis, and hydrophobia. The diseases in Cluster 1 never occur or rarely occur in South Korea. For instance, from January 2016 to October 2019, hydrophobia was never reported. In the case of cholera, most of the patients were reported in 2018 with one or two patients in each region. Similarly, Cluster 2 contained rare diseases, such as measles, rubella, and Q fever. The difference between Cluster 1 and Cluster 2 was that the diseases in Cluster 1 had similar occurrence patterns in most regions, whereas those in Cluster 2 did not. Figure 7a,b presents the normalized occurrences of Japanese encephalitis in Cluster 1 and measles in Cluster 2, respectively. Japanese encephalitis periodically occurred in a few regions every July to October. In contrast, measles occurred irregularly in some regions from 2016 to 2018, and the number of patients suddenly increased in 2019. Meanwhile, Japanese encephalitis seemed to have patterns similar to severe fever with thrombocytopenia syndrome of Cluster 5 illustrated in Figure 7e. However, Japanese encephalitis was assigned to Cluster 1 rather than to Cluster 5 due to the relatively small number of reported cases.

Cluster 3 consisted of infectious diseases whose patients continue to be reported in most regions without seasonality. Figure 7c illustrates the normalized occurrences of acute hepatitis B in Cluster 3. Patients with acute hepatitis B were present consistently in all regions. However, the peak points in each region were slightly different from each other, which results in a relatively complicated graph, as shown in Figure 7c. Hepatitis A showed similar patterns except that the peak points of all regions appeared only in early 2016 or at the end of 2019.

Cluster 4 comprises three infectious diseases commonly categorized as “febrile illness during the fall” in South Korea. Figure 7d reveals the normalized occurrences of scrub typhus. The number of patients with the disease drastically increased during fall and early winter, and afterward, decreased rapidly. This occurrence pattern repeated every year, and the other infectious diseases in this cluster exhibited similar trends. Meanwhile, leptospirosis demonstrated both a pattern of Cluster 4 and a pattern similar to Cluster 5, presented in Figure 7e. This is because patients with leptospirosis were often reported during late summer.

Cluster 5 comprises infectious diseases frequently reported from spring to fall. The patients continued to develop diseases during that period, with a few cases reported in winter. Figure 7e presents the normalized occurrences of severe fever with thrombocytopenia syndrome, which illustrates that trend. Among the four diseases in Cluster 5, patients with enterohemorrhagic *Escherichia* *coli* infection had been reported more frequently in winter compared to the other diseases. Thus, its occurrence pattern was somewhat similar to that of Cluster 3.

Finally, Cluster 6 was composed of infectious diseases whose periodicity was roughly six months. The number of patients increased during the transition period from spring to summer and from fall to winter, with a similar trend in most regions. Chickenpox showed that trend perfectly, as shown in Figure 7f. Although pneumococcus and primary syphilis also showed such a trend, their peak points in each region were different from each other, and the peak-to-peak differences were relatively marginal, unlike those of chickenpox.

### 3.3. Analysis of Time-Based Clustering

**Figure 8:**
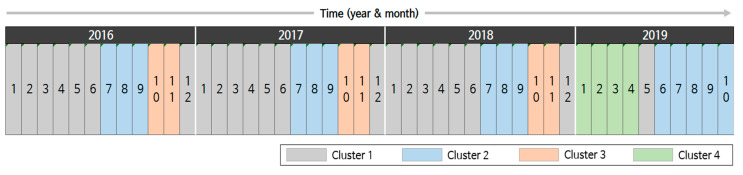
Clustering results by time.

Time is another important clustering criterion. To observe the temporal trend, we performed month-based clustering based on the disease occurrence pattern. Figure 8 depicts the results for 46 months from January 2016 to October 2019 where four clusters are represented using assorted colors. In the figure, Clusters 1 to 3 repeat from January 2016 to December 2018. More specifically, Clusters 1, 2, and 3 repeated from winter to early summer, in summer, and in fall, respectively. This tendency indicates that infectious diseases, such as scrub typhus and influenza, have seasonality [12,42]. When we considered the clusters obtained in the disease-based clustering, we could roughly connect the disease clusters and time clusters. For instance, diseases in disease Cluster 3 are closely related to those in time Cluster 3. Second, diseases in disease Clusters 5 and 6 were determinants of time Clusters 1 and 2.

However, the trends changed dramatically in 2019. A new cluster, Cluster 4, appeared in January 2019, which had not been previously observed. Cluster 4 spanned until April, and then, Cluster 1 appeared in May 2019. However, in the next month, Cluster 2 started, which was one month earlier than in the previous years. Further, Cluster 2 lasted for 5 months. This indicates that the pattern of infectious disease occurrences considerably changed in 2019. Such changes can be explained by investigating the main diseases and their occurrences.

**Figure 9:**
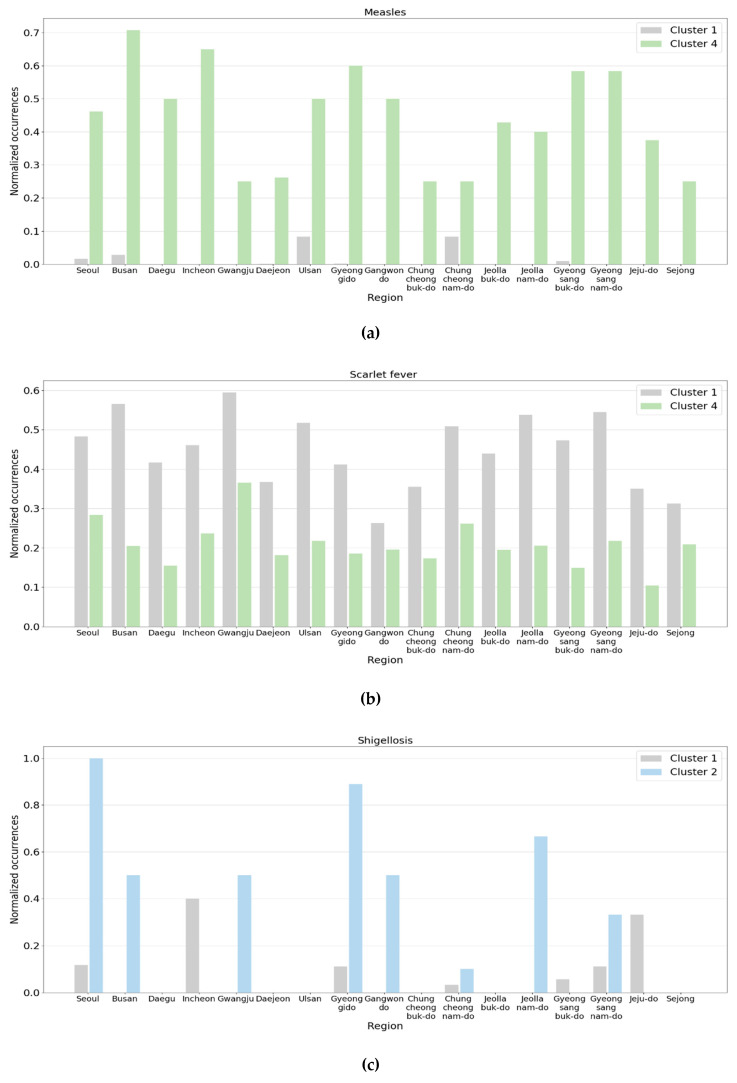

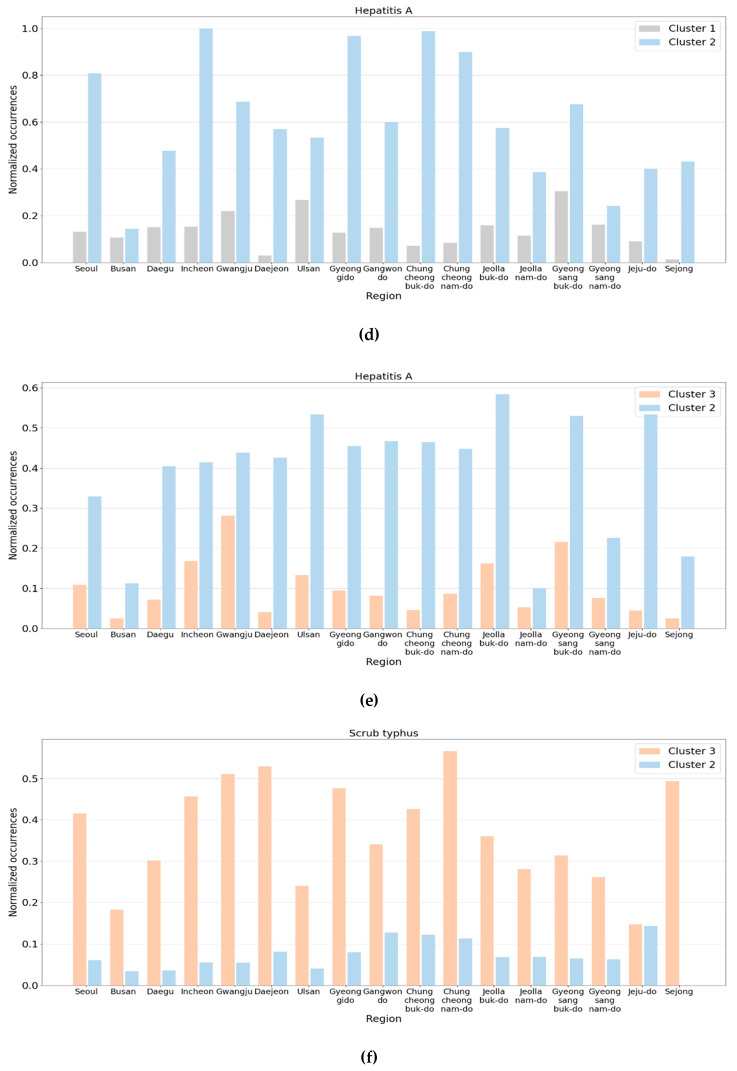
Comparison of normalized occurrences of infectious diseases between clusters: (**a**) measles in Clusters 1 and 4; (**b**) scarlet fever in Clusters 1 and 4; (**c**) shigellosis in Clusters 1 and 2; (**d**) hepatitis A in Clusters 1 and 2; (**e**) hepatitis A in Clusters 3 and 2; (**f**) scrub typhus in Clusters 3 and 2.

Figure 9 presents some pairs of clusters where noticeable changes were observed in the normalized occurrences. In the graphs, the *x*-axis and *y*-axis represent the region and normalized occurrence, respectively, and the clusters were represented using the colors defined in Figure 8. Figure 9a illustrates the normalized occurrences of measles of Clusters 1 and 4. The average number of patients with measles was about 50 per month in early 2019 and increased to 260 in April. It was about two per month before 2019. In May 2019, the number of patients decreased and went back to the trend from Cluster 1. In the figure, the normalized occurrences of Cluster 1 were so small that the maximum among the regions was only about 0.1. However, all the normalized occurrences of Cluster 4 ranged from 0.3 to 0.7.

Another major change is the reduction in patients with scarlet fever. From January to April 2019, the reported cases of scarlet fever nationwide decreased by more than half of those reported in the same month in 2018, and in a few regions, the number was reduced to a quarter. This is shown in Figure 9b. In Figure 9b, we observe that the normalized occurrences of Cluster 1 were equal to or more than about twice those of Cluster 4 in most regions.

The second pair of clusters we investigated was Clusters 1 and 2. The total number of patients with shigellosis nationwide increased slightly in June 2019. Although the number was only about 20, it was nearly three times larger than the previous year. Further, the number of patients with hepatitis A drastically increased, which reached about 20 times the patient number in the previous year. Figure 9c,d reveals the normalized occurrences of shigellosis and hepatitis A, respectively. In Figure 9c, the normalized occurrences of Cluster 1 were close to zero except for a few regions. However, in June 2019, some regions had considerably large values compared to Cluster 1. Meanwhile, the differences between the two clusters in Figure 9d were more noticeable compared to those in Figure 9c.

Lastly, the number of patients with hepatitis A and measles increased, but the number of patients with scarlet fever decreased in October 2019, compared to the same month of the previous year. In addition, the number of patients with scrub typhus that frequently occurred in the fall declined significantly in 2019. This is presumed to be the primary reason October 2019 was assigned to Cluster 2 instead of Cluster 3. Figure 9e,f displays the normalized occurrences of hepatitis A and scrub typhus, respectively. In the figure, although the normalized occurrences of Cluster 3 were much lower than those of Cluster 2, the normalized occurrences of Cluster 3 were much larger than those of Cluster 2.

### 3.4. Analysis of Region-Based Clustering

**Figure 10:**
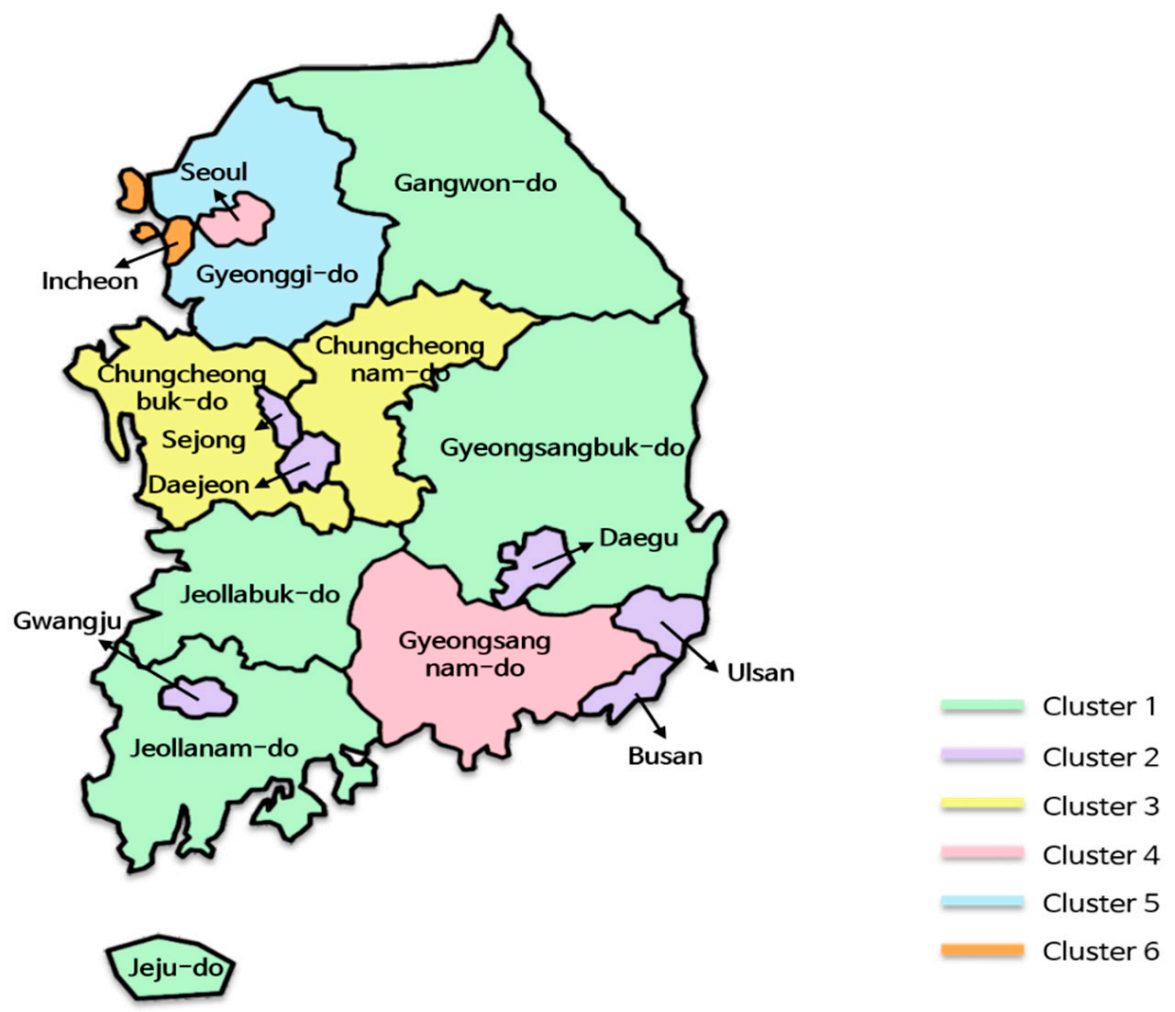
The region clusters.

The last clustering that we performed was in regions. Figure 10 reveals the results where six region clusters were created from 17 regions, represented using different colors. Generally, geographically adjacent regions were more likely to be grouped into the same cluster. For instance, Cluster 3 consisted of two adjacent regions, Chungcheongbuk-do and Chungcheongnam-do, and Cluster 1 comprised spacious and connected regions, such as Gangwon-do, Gyeongsangbuk-do, Jeollabuk-do, and Jeollanam-do. Although Jeju-do is not directly connected because it is an island, it was included in Cluster 1. Meanwhile, metropolises, such as Busan, Daegu, Ulsan, Gwangju, Daejeon, and Sejong, except for Incheon and Seoul, were grouped into Cluster 2 regardless of their locations. This result is consistent with previous studies, in that the degree of urbanization led to differences in the occurrence patterns of the infectious diseases [43,44].

However, we did not find significant differences between clusters, unlike the disease-based clusters or time-based clusters. That is, the difference in the occurrence of infectious diseases was too small to separate into clusters. We assume that this is because South Korea has a well-developed transportation system and is a small territory compared to other countries, such as the United States, Canada, and China. As the exchange of people between regions is active, regions tend to have similar characteristics in terms of disease occurrences.

One notable point is that Seoul belongs to Cluster 4 along with Gyeongsangnam-do. Although Seoul is the biggest metropolis in Korea, it was not grouped with other metropolises, such as Incheon and Gyeonggi-do, which are close to Seoul. Just by comparing the number of patients or normalized occurrences of regions, we could not find any significant difference between clusters. However, by constructing tensors from the extracted features of regions listed in Table 4 and comparing them, we observed some differences between the tensors. Using this methodology, we found that the normalized occurrences were roughly dependent on feature F1 in Table 4. From this viewpoint, Seoul (0.331) had an occurrence pattern that was more similar to Gyeonggi-do (0.337) than to Gyeongsangnam-do (0.261) for various infectious diseases, including hepatitis A, chickenpox, and scrub typhus. However, considering the rest of the features, Seoul and Gyeongsangnam-do formed a cluster (Cluster 4), and Gyeonggi-do formed another cluster (Cluster 5).

In Cluster 2, the features of Sejong were different from those of other regions. In particular, Sejong exhibited significant differences in F1 and F2 compared to the other regions in the cluster. For instance, the F1 and F2 values of Sejong were 0.132 and −0.587, respectively, while their cluster averages were 0.211 and −0.165, respectively. Hence, when we performed *k*-means clustering with *k* set to 7, Sejong formed a new cluster, and other clusters remained unchanged. When we investigated the occurrences of diseases in Sejong, we found that overall infectious disease occurrences in this city were fewer than that in the other cities. We think this is because Sejong has the smallest population among the regions. Jeju-do, which has the second smallest population, also had a similar F1 value.

## 4. Conclusions

In this paper, we proposed a clustering-based analysis scheme for investigating the occurrence patterns of infectious diseases. To do this, we collected disease occurrence data containing time, region, and infectious disease and constructed a tensor. Then, we extracted latent features from the tensor by using Tucker decomposition and performed *k*-means clustering for each dimension in the latent spaces. To demonstrate the effectiveness of the scheme and how to interpret the obtained results, we conducted a case study of South Korea and showed the resulting clusters for each dimension. We analyzed the results by comparing the raw data and extracted features. Some disease clusters had seasonality and periodicity, whereas other disease clusters showed an aperiodic occurrence pattern. Further, we explained the changes in disease occurrences over time. We observed the abrupt changes between 2019 and the previous years and derived the reason from the data. Lastly, we confirmed the differences in the occurrence patterns between region clusters, caused by the degree of urbanization and geographical adjacency.

In the future, we aim to extend our scheme on a global scale to analyze infectious disease occurrence patterns affecting a wide range of countries. In addition, we will investigate deep learning-based feature extraction methods to extract better features of the given data and use explainable artificial intelligence techniques for a more effective explanation of the analysis results.

## Figures and Tables

**Table 1:** A list of infectious diseases.

#	Infectious Disease	#	Infectious Disease
1	Cholera	29	Paratyphoid fever
2	Enterohemorrhagic *Escherichia* *coli* infection	30	Diphtheria
3	Tetanus	31	Mumps
4	Polio	32	Chickenpox
5	*Haemophilus influenzae* type b	33	Malaria
6	Leprosy	34	Legionellosis
7	Epidemic typhus	35	Scrub typhus
8	Brucellosis	36	Hydrophobia
9	Primary syphilis	37	Congenital syphilis
10	Plague	38	Dengue fever
11	Smallpox	39	Severe acute respiratory syndrome
12	Novel swine-origin influenza A(H1N1)	40	Q fever
13	Lyme disease	41	Melioidosis
14	Emerging infectious diseases	42	Middle East respiratory syndrome coronavirus
15	Typhoid fever	43	Shigellosis
16	Hepatitis A	44	Pertussis
17	Measles	45	Rubella
18	Japanese encephalitis	46	Acute hepatitis B
19	Pneumococcus	47	Scarlet fever
20	*Neisseria meningitidis*	48	*Vibrio vulnificus* sepsis
21	Murine typhus	49	Leptospirosis
22	Anthrax	50	Hemorrhagic fever with renal syndrome
23	Secondary syphilis	51	Creutzfeldt–Jakob disease (CJD)/variant CJD
24	Yellow fever	52	Viral hemorrhagic fevers
25	Botulinum toxin	53	Avian influenza
26	Tularemia	54	West Nile fever
27	Tick-borne viral encephalitis	55	Chikungunya fever
28	Severe fever with thrombocytopenia syndrome	56	Zika virus infection

**Table 2:** A list of regions.

#	Region	#	Region
1	Seoul	10	Chungcheongbuk-do
2	Busan	11	Chungcheongnam-do
3	Daegu	12	Jeollabuk-do
4	Incheon	13	Jeollanam-do
5	Gwangju	14	Gyeongsangbuk-do
6	Daejeon	15	Gyeongsangnam-do
7	Ulsan	16	Jeju-do
8	Gyeonggi-do	17	Sejong
9	Gangwon-do		

**Table 3:** A list of hyperparameters.

Variable	Description	Value
*N_R_*	The number of elements in the region dimension	17
*N_I_*	The number of elements in the infectious disease dimension	56
*N_T_*	The number of elements in the time dimension	46
*d_R_*	The number of latent features in the region dimension	4
*d_I_*	The number of latent features in the infectious disease dimension	4
*d_T_*	The number of latent features in the time dimension	4
*k_R_*	The number of clusters in the region dimension	6
*k_I_*	The number of clusters in the infectious disease dimension	6
*k_T_*	The number of clusters in the time dimension	4

**Table 4:** Extracted features of the regions.

Cluster	Region	Features			
		F1	F2	F3	F4
1	Gangwon-do	0.218	−0.153	0.269	−0.212
	Gyeongsangbuk-do	0.258	−0.065	0.285	−0.233
	Jeollabuk-do	0.234	−0.256	0.147	−0.210
	Jeollanam-do	0.216	−0.139	−0.045	−0.392
	Jeju-do	0.177	−0.117	−0.182	−0.335
	Average	0.220	−0.146	0.095	−0.277
2	Daejeon	0.216	−0.047	0.004	0.193
	Daegu	0.243	−0.044	−0.162	−0.034
	Gwangju	0.201	−0.176	−0.098	0.238
	Busan	0.257	−0.044	−0.327	−0.127
	Ulsan	0.214	−0.093	−0.392	0.314
	Sejong	0.132	−0.587	−0.192	0.136
	Average	0.211	−0.165	−0.194	0.120
3	Chungcheongbuk-do	0.205	−0.098	0.256	0.250
	Chungcheongnam-do	0.261	−0.187	0.396	0.186
	Average	0.233	−0.143	0.326	0.218
4	Seoul	0.331	0.305	−0.003	0.352
	Gyeongsangnam-do	0.261	0.201	−0.178	0.184
	Average	0.296	0.253	−0.091	0.268
5	Gyeonggi-do	0.337	0.420	0.318	−0.025
6	Incheon	0.276	0.359	−0.319	−0.326
