# Erratum: Jung, S., et al. Cluster-Based Analysis of Infectious Disease Occurrences Using Tensor Decomposition: A Case Study of South Korea. *Int. J. Environ. Res. Public Health* 2020, *17*, 4872

**DOI:** 10.3390/ijerph17249493

**Published:** 2020-12-18

**Authors:** Seungwon Jung, Jaeuk Moon, Eenjun Hwang

**Affiliations:** School of Electrical Engineering, Korea University, 145 Anam-ro, Seongbuk-gu, Seoul 02841, Korea; jsw161@korea.ac.kr (S.J.); jaewookmo@korea.ac.kr (J.M.)

The source of funding should be modified in our original article [1]. Therefore, in the section “Funding”, where it is stated: “This research was supported by a grant of the Korea Health Technology R&D Project through the Korea Health Industry Development Institute (KHIDI), funded by the Ministry of Health and Welfare, Republic of Korea (HG19C0682).”, we would like to add the following information:
